# Automatic Classification and Acoustic Auscultation of Heart, Lung, and Bowel Sounds Using Artificial Intelligence

**DOI:** 10.21203/rs.3.rs-7061625/v1

**Published:** 2025-07-29

**Authors:** Yen-Sheng Lin, Ansh Kapadia, Eric B. Ortigoza

**Affiliations:** 1 Department of Orthopaedic Surgery, UT Southwestern Medical Center, Dallas, TX; 2 Department of Physical Medicine and Rehabilitation, UT Southwestern Medical Center, Dallas, TX; 3 Texas Academy of Mathematics and Science, University of North Texas, Denton, TX; 4 Division of Neonatal-Perinatal Medicine, Department of Pediatrics, UT Southwestern Medical Center, Dallas, TX

**Keywords:** Artificial Intelligence, Acoustic Auscultation, Automated Algorithm

## Abstract

Auscultation of heart, lung, and bowel sounds remains a fundamental diagnostic technique in clinical practice despite significant technological advancements in medical imaging. However, the accuracy of auscultation-based diagnoses is highly dependent on clinician experience and expertise, leading to potential diagnostic inconsistencies. The objective of this study is to present a novel artificial intelligence (AI) framework for the automatic classification and acoustic differentiation of heart, lung, and bowel sounds, addressing the need for objective, reproducible diagnostic support tools. Our approach leverages recent advances in supervised machine learning and signal processing to extract distinctive acoustic signatures from publicly available, digitized heart, lung, and bowel sounds. By analyzing spectral, temporal, and morphological features across diverse asymptomatic populations, the algorithm achieves excellent classification of predictive accuracy (65.00% to 91.67%) and validation accuracy (83.87% to 94.62%) from six AI models. The clinical implications of this algorithm show promise beyond diagnostic support to applications in medical education, telemedicine, and continuous patient monitoring. This work contributes to emerging AI-assisted auscultation by providing a comprehensive framework for multi-organ sound classification with the potential to improve differential diagnostic accuracy and standardization in clinical settings.

## Introduction

Auscultation of heart, lung, and bowel sounds (HLBS) remains a fundamental diagnostic technique in clinical practice despite significant technological advancements in medical imaging [1]. This examination is non-invasive and uses a clinically standard approach to evaluate cardiovascular, respiratory, and abdominal diseases that produce pathological acoustic signals. However, the accuracy of auscultation-based diagnoses is highly dependent on clinician experience and expertise, leading to potential diagnostic inconsistencies [2]. Therefore, the study of HLBS methods for objective classification and differentiation is of great clinical significance as the first step to improve differential diagnosis. The objective of this study is to present a novel artificial intelligence (AI) framework for the automatic classification and differentiation of HLBS, addressing the need for objective, reproducible diagnostic support tools.

The transparent process of feature selection in acoustic signal classification is one of the most important steps needed to build accurate, interpretable, and explainable machine learning models. Specific acoustic feature selection methods fall into one of three categories: filter, wrapper, and embedded methods. Identifying and eliminating irrelevant features allows for more efficient acoustic classification and differentiation models with improved accuracy, interpretability, and explainability. No single feature selection fits all applications. There is a trade-off between the filter and wrapper methods: filter methods are computationally simpler while wrapper methods outperform for large datasets [3]. Recent advancements in signal processing technology and deep learning allow for more sophisticated analysis of digitized heart sounds [4–6]. Acoustic features of heart sounds can closely represent the characteristics of heart disease [5]. Vibrations caused by cardiac activities such as myocardial contraction, heart valve closure, and occlusion of the ventricular wall are transmitted through the tissue to the surface of the chest wall and form the heart sound signals. Therefore, this phenomenon can be perceived by the human ear and recorded with electronic instruments. According to the order of occurrence in a cardiac cycle, the heart sound is divided into four components: the first heart sound (S1), the second (S2), the third (S3), and the fourth (S4). The intensity, frequency and correlation of the heart sounds reflect the heart valve condition, myocardial function, and intracardiac blood flow [7]. Significant advancements in lung sound analysis include deep learning-based algorithms for the detection and classification of various respiratory conditions [8]. Lung sounds are categorized as either normal (vesicular, bronchial, brocho-vesicular, or tracheal) or abnormal/aberrant or adventitious lung sounds. Depending on their duration, the adventitious lung sounds can either be continuous or discontinuous. Adventitious lung sounds are commonly referred to as wheezing, crackling, rhonchi, or stridor, and their presence often indicates pulmonary pathology [9–11]. The analysis of bowel sounds, while less extensively studied compared to heart and lung sounds, is of growing interest. Researchers have investigated the correlation between bowel sounds and gastrointestinal motility and have explored automated methods for detecting and classifying different types of bowel sounds [12]. Several studies have combined the HLBS analysis with multi-modal approaches aimed to provide a more comprehensive assessment of a patient’s condition by simultaneously analyzing real-time physiological signals [13–15].

Despite advancements of multi-modal approaches, noise interference, inter-patient variability, and the need for large, diverse datasets are common challenges in creating robust, generalizable models that can accurately classify various types of biological sounds. While heart, lung, and bowel sounds have each been studied individually, an integrated approach to HLBS classification remains underexplored. In this study, we focused on identifying the most accurate acoustic feature selection method using supervised machine learning algorithms for HLBS classification and differentiation.

## Methods

**Figure 1.**
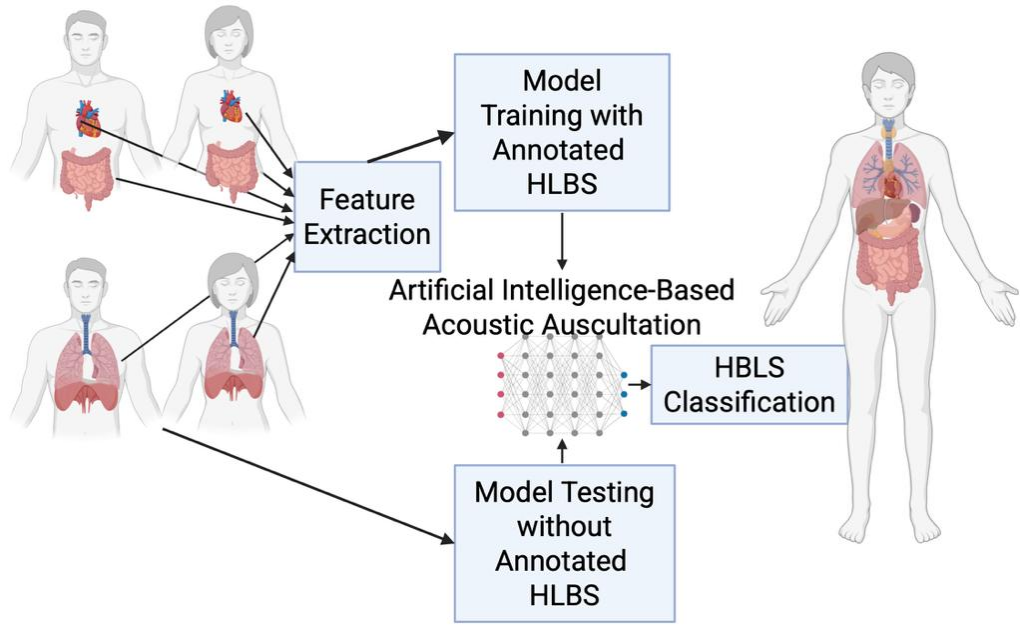
Heart, lung, and bowel sound (HLBS) classification through artificial intelligence-based acoustic auscultation algorithms.

Our approach leverages recent advances in supervised machine learning, deep learning, and signal processing to extract distinctive acoustic signatures from digitally recorded physiological sounds. Pre-recorded HLBS contain distinctive acoustic features that can be extracted and used to classify unlabeled audio samples containing these sounds. The steps involved in this classification include the preprocessing of HLBS signals, feature extraction, data processing, algorithm training, model testing, and real-world application (Figure 1). In this study, a novel framework was proposed utilizing publicly available acoustic datasets to develop feature selection methods from filter, wrappers, and embedded algorithms [16–18]. The performance of each of the six supervised machine learning-based models were analyzed using metrics such as accuracy, sensitivity, specificity, and validation accuracy (Support Vector Machines, Neural Networks, Random Forests, K-Nearest Neighbor Algorithms, Decision Trees, and Naive Bayes Classifiers) to assess the model performance.

### Acoustic Data Sources and Pre-Processing

**Figure 2.**
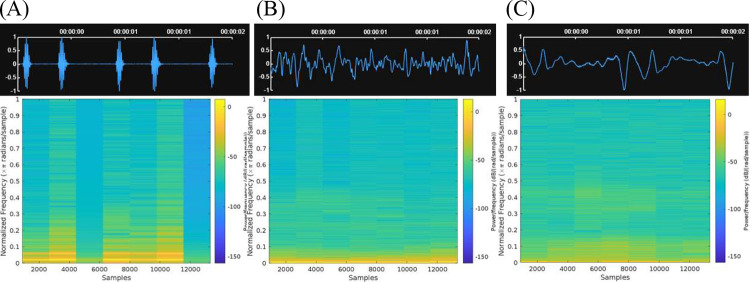
The waveforms and spectrograms of typical noise and heart (2A), lung (2B), and bowel (2C) sound segments.

A total of 120 heart, 81 lung, and 123 bowel sounds were obtained from publicly available datasets [16–18]. Heart sounds were recorded using an electronic stethoscope and samples at 8kHz. Lung sounds were recorded using an electronic stethoscope from 126 patients and sampled at 10 kHz. Bowel sounds were recorded using intestinal sound-dedicated contact microphone from 19 subjects and sampled at 44.1 kHz. For this study, out of all the available sound files in the database, only normal HLBS were used. Each audio file was sliced into 2-second segments containing heart, lung or bowel sounds. This allows capturing the relevant acoustic features while maintaining manageable data size. It also allows models to focus on code characteristics of heart, lung, and bowel sounds without being influenced by extraneous factors such as variable recording lengths or silence. To avoid unnecessary computational power in future processes, all recordings were down sampled to 8 kHz. Figure 2 demonstrates the waveforms and corresponding spectrograms for HLBS.

### Feature Extraction

Heart, lung, and bowel sounds all have unique characteristics. Heart sounds usually have a regular pattern corresponding to S1 and S2 sounds. Lung sounds are more continuous and varied. Bowel sounds in comparison are usually more intermittent with irregular and varied patterns. Multiple prior studies have extracted acoustic features from HLBS signals such as discrete wavelet transform (DWT) features, continuous wavelet transformation (CWT) features, short-time Fourier transform (STFT) features, and Mel Frequency Cepstral Coefficients (MFCC). However, the most widely used algorithms are the MFCC for computational efficiency and effectiveness to capture spectral envelopes and the DWT for analyzing both temporal and spectral audio signatures. We employed a multi-domain approach for feature extraction to comprehensively capture the unique characteristics of each sound type. In this study we extracted the following features: Time domain features (mean peak distance, envelop peak ratio, zero crossing rate), frequency domain features (frequency ratio, spectral entropy), time-frequency domain features (wavelet energy), and cepstral domain features (mel-frequency cepstral coefficients).

### Mel-Frequency Cepstral Coefficients (MFCC)

These are coefficients that collectively represent the short-term power spectrum of a sound, based on a linear cosine transform of a log power spectrum on a nonlinear mel scale of frequency. The extraction of these data required 4 steps: 1) Apply Short-Time Fourier Transform with a window size of 32ms and 0.5x overlap. 2) Map the powers of the spectrum onto a mel scale. 3) Take the logs of the powers at each of the mel frequencies. 4) Apply the discrete cosine transform. Each of the audio files were mapped to the 124 MFCC features (13 features per window) generated from their signal data. Each audio file had a mean peak distance feature, a frequency ratio feature, an envelope peak ratio, 5 wavelet energy features, a spectral entropy feature, a zero-crossing rate feature, and 124 MFCC features. The extracted features were placed into a feature matrix, where each row corresponded with 1 audio file, and each column represented 1 feature. Signal processing methods were used for identification and extraction of large feature sets from acoustic signals and enabled automatic detection of HLBS.

### Data Processing

20 HS, LS and BS were kept separate as a test dataset for unbiased blinded evaluation of the models once they are created. Remaining audio files underwent Synthetic Minority Oversampling Technique (SMOTE) procedure before dividing it into Training (70%) and validation set (30%). We used SMOTE to address imbalance in the training set that may potentially result in a low classification accuracy. We obtained 61 high quality normal lung sounds, compared to normal heart sounds (n=100) and normal bowel sounds (n=103). We used SMOTE to create more samples of the minority class while avoiding overfitting. This is done by generating new synthetic samples that are close to the other points belonging to the minority class in the feature matrix. To ensure all features were on a common scale, the feature matrix was normalized. The mean and standard deviation used during normalization were saved for later use. Next, we implemented feature selection that implements five different methods to identify and remove features with: more than 60% missing values, collinear variables with 0.995 correlation, 0.0 ‘importance’ to the data, only a single unique value, and low importance features that do not contribute to 0.99 cumulative feature ‘importance’[19]. The importance of each feature was decided by a gradient boosting regression model.

### Supervised machine learning models

We implemented six machine learning algorithms to classify HLBS signals. The SVM was configured with a linear kernel without optimizing hyperparameters. The neural network architecture consisted of one fully connected layer with 10 neurons trained with an Adam optimizer. The k-Nearest Neighbor (kNN) algorithm was implemented with k = 1. For the decision tree classifier, we performed the model with the default minimum sample per leaf without the restrictions on maximum depth-to-balance model complexity and generalization. The Naïve Bayes implementation used kernel distribution assumption for continuous acoustic features after verifying feature independence assumptions. Finally, the Random Forest created a bagging ensemble through decision trees with bootstrapping sampling, with each tree considering a random subset of all features at each split, thereby improving model robustness against noise artifacts in acoustic recordings. We used a symmetric cost matrix assigning a penalty of 1 to all misclassifications and 0 to correct predictions, enabling the model to treat all class errors equally.

### Performance evaluation

Performance of each supervised machine learning were assessed by sensitivity, specificity, and accuracy, which were calculated to evaluate the performance of each supervised machine learning classifier. Sensitivity and specificity are defined as: Sensitivity = TP/(TP+FN) and Specificity = TN/(TN+FP). Where the true positives (TP) are the numbers of the samples derived from each HLBS segment that are correctly identified, and true negatives (TN) are samples derived from segments that are predicted as noise. False positives (FP) and false negatives (FN) are the corresponding false predictions. When testing the accuracy of the developed classifier for each HLBS type, the accuracy is defined as the proportion of samples from each HLBS subtype group that are correctly identified. We evaluated the classification performance based on the macro-averaged F1 score as a primary metric due to its effectiveness in handling class imbalance. For each HLBS category, precision was computed as the ratio of true positives to the sum of true positives and false positives. To obtain the macro-averaged F1 score, we calculated the unweighted arithmetic mean of the F1 scores across all classes, giving equal importance to each category regardless of its frequency in the dataset. This approach provided a balanced performance assessment that accounted for the natural variation of HLBS, ensuring that the effectiveness of our AI models was not overstated due to high performance on majority classes.

## Results

**Figure 3.**
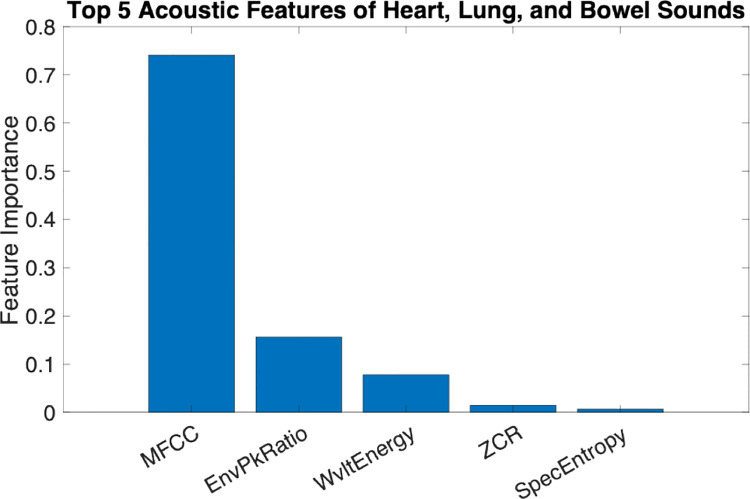
Top 5 features of heart, lung, and bowel sounds.

**Figure 4.**
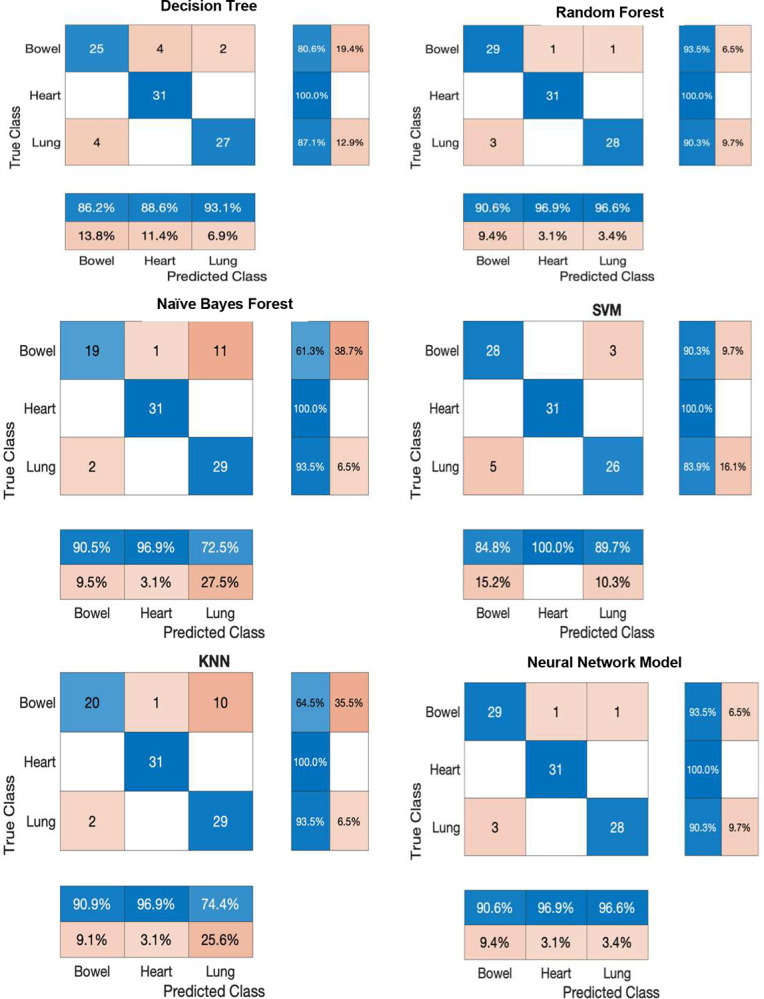
Confusion Matrix for the 6 models to predict the blinded independent set of acoustic sources. The true positives are the diagonal elements. False positives represent the top off-diagonal while the false negatives represent the elements bottom off-diagonal. A column-normalized column summary displays the number of correctly (top row) and incorrectly (bottom tow) classified observations for the predicted HLBS as percentages of the number of observations of the corresponding predicted class. A row-normalized row summary displays the number of correctly (left column) and incorrectly (right column) classified observations for true HLBS as percentages of the number of observations of the corresponding true class.

Our results highlight the relative importance of each acoustic feature, with MFCCs emerging as the most powerful discriminative feature (72.86% contribution), followed by Envelope Peak Ratio (15.99%), Wavelet Energy (9.33%), Zero Crossing Rate (0.99%), and Spectral Entropy (0.49%) (Figure 3). The performance of six supervised machine learning models using the training dataset are listed in Table 1. Among these classifiers, Neural Network based Model showed the highest accuracy, sensitivity, specificity, precision on par with the Random Forest model. In Table 2, the highest accuracy (94.62%) and F1 score (0.95) of Neural Network based model outperformed the other five classifiers using the testing data set. However, the computation performance is about three-fold increase compared to the Decision Tree model which has the lowest accuracy (83.87%) and F1 score (0.84) but with the highest computation performance (0.03 secs). The predictive accuracy and computation performance for different HLBS types is listed in Table 3. While MFCCs alone provided a strong baseline, integrating all five features produced the highest classification accuracy showing ultimate performance gains. After testing six supervised machine learning algorithms, all demonstrated high sensitivity and specificity for HLBS classification (>84%), with SVM achieving the highest predictive accuracy (91.67%). Validation accuracy was the highest for SVM, Neural Network-based model, and Random Forest. The confusion matrix (or blinded independent test set) further confirmed the robustness of these algorithms (Figure 4). Among them, SVM was the most sensitive and specific for HLBS classification.

## Discussion

In this study, we developed and evaluated an AI-based framework for the automatic classification and acoustic differentiation of heart, lung, and bowel sounds. The proposed model demonstrated promising performance for future use in distinguishing among normal and pathological sounds across multiple organ systems. The supervised machine learning approaches achieved classification accuracy of 100% for heart sounds, 87.1% to 93.5% for lung sounds, and 61.3% to 93.5% for bowel sounds, representing a significant advancement in computer-aided auscultation technology. For predictive accuracy, the model achieved 88.6% to 100% for heart sounds, 72.5% to 96.6% for lung sounds, and 86.2% to 90.9% for bowel sounds. These findings highlight the feasibility and potential of AI-enhanced auscultation as a non-invasive, accessible, and efficient diagnostic tool. The integration of both time and frequency features in our model appears to be particularly important for bowel sound analysis, an area that has received comparatively less attention in the literature. Previous studies by Thompson et al. focused primarily on detection rather than classification of bowel sounds, achieving 78% sensitivity in identifying bowel activity with averages of classification and predictive accuracy of 77.4% and 88.6%, respectively. Although the dataset did not include the different patterns of adult bowel sounds, future works are warrantied to implement the prospective study to develop the disease-specific dataset to refine the algorithms for testing our own classification and predictive accuracy of HBLS.

A key strength of our approach is the comprehensive preprocessing pipeline, which effectively reduces environmental noise and enhances the signal characteristics specific to each anatomical sound source. The MFCC technique proved particularly effective for detecting transient events in HLBS, while our novel application captured the temporal dynamics of bowel sounds with greater fidelity than previous methods [20]. MFCCs emerged as the post powerful discriminative feature of HLBS. The analysis also demonstrated how all features (MFCCs, Envelope Peak Ratio, Wavelet Energy, Zero Crossing Rate, and Spectral Entropy) complement each other, with each capturing different aspects of the physiological sounds.

The potential clinical applications of this AI-based technology are substantial and translational. Our system could serve as a training tool for medical students and residents, providing objective feedback on auscultation technique and interpretation. In addition, it could function as a decision support system for clinicians, particularly in primary care or resource-limited settings where specialist consultation may not be readily available. Moreover, this AI-based technology could enable tele-health with remote auscultation and monitoring, allowing patients to perform self-examinations at home with results interpreted automatically or reviewed by healthcare providers. The high sensitivity and specificity of our model in distinguishing between multi-organ conditions provides a framework for a transparent and explainable process for AI-based auscultation.

This study has several limitations. Our training dataset, while diverse, may not fully represent the spectrum of acoustic variations present in the populations across the lifespan with different body habitus, ages, and comorbidities. In addition, the performance of our algorithms in real-world clinical environments with ambient noises may need to be tested with various noise cancelling capacities. The datasets were based on the adult population without known pathologies in HLBS, which may limit the generalizability of the algorithms to the pathological or pediatric population across the lifespan. While the database included recordings from various stethoscope models, the acoustic properties of different models of stethoscope will need future validation to include various acoustic sources for the generalizability. The computation performance varies in selected supervised models, which may require the parameter-efficient fine-tuning process to update only a subset of the parameters to reduce the computational costs. In addition, the challenge of obtaining accurately annotated training data deserves special mention. Unlike medical imaging, where expert consensus on visual findings is often achievable, auscultation findings can be subjective among clinicians. The methodology consensus will provide the ground truth annotated training data with comparison between independent clinicians and AI-based algorithms for clinical validation.

## Conclusions

In this study, we have demonstrated a supervised machine learning benchmark specifically designed to automate HLBS classification. This innovation is particularly significant for precision medicine and tele-health applications, enabling automatic characterization of complex acoustic signatures during auscultation. A key contribution of our study is the knowledge propagation algorithm in supervised machine learning designed to mitigate the acoustic information loss by ensuring the crucial features are not discarded during the learning process. This allows each model to learn robust and generalizable representations, leading to more accurate predictions. In addition, our models incorporate only highly distinctive features to introduce dimension reductionality while preserving key aspects of different physiological sounds. The experimental results support the effectiveness of our supervised models and achieve a new state-of-art performance. A notable strength of our models are their pronounced accuracy, high sensitivity and specificity to identify HLBS, a critical requirement for effective tele-health management. These results confirmed the potential of our approach as a robust tool in the field of acoustic health diagnostics. Further research is warranted to expand the dataset to include pediatric, neonatal, and pathological recordings and to explore real-time implementation for bedside and telehealth integration, especially in vulnerable populations.

## Figures and Tables

**Table 1.** Performance of six supervised machine learning classifiers using the training dataset

	Accuracy (%)	Sensitivity (%)	Specificity (%)	Precision (%)	Macro-averaged F1 Scores	Matthews Correlation Coefficient	Cohen’s Kappa
Neural network	94.62%	94.62%	97.31%	94.68%	94.60%	91.98%	87.90%
KNN	86.02%	86.02%	93.01%	87.38%	85.58%	79.86%	68.55%
SVM	91.40%	91.40%	95.70%	91.50%	91.39%	87.16%	80.65%
Naïve Bayes	84.95%	84.95%	92.47%	86.62%	84.39%	78.42%	66.13%
Random Forest	94.62%	94.62%	97.31%	94.68%	94.60%	91.98%	87.90%
Decision Tree	89.25%	89.25%	94.62%	89.29%	89.09%	83.96%	75.81%

**Table 2:** Accuracy of six supervised machine learning classifiers using validation data set

Type	Accuracy (%)	F1 Score	Computation Performance (secs)
**Neural network-based model**	94.62%	0.95	0.09
**SVM**	92.47%	0.93	0.06
**Random Forest**	92.47%	0.93	0.14
**KNN**	89.25%	0.89	0.07
**Naïve Bayes**	86.02%	0.88	0.19
**Decision Tree**	83.87%	0.84	0.03

**Table 3.** Predictive Accuracy of supervised machine learning classifiers for six types of models using testing dataset (unbiased evaluation)

Type	Predictive Accuracy (%)	F1 Score	Computation Performance (secs)
**SVM**	91.67%	0.92	0.020
**KNN**	81.67%	0.82	0.019
**Neural network-based model**	78.33%	0.79	0.022
**Random Forest**	75.00%	0.80	0.063
**Naïve Bayes**	73.33%	0.74	0.133
**Decision Tree**	65.00%	0.68	0.007

## Data Availability

Heart sound audio files were obtained from Database: https://github.com/yaseen21khan/Classification-of-Heart-Sound-Signal-Using-Multiple-Features-/blob/master/README.md. Lung sounds audio files were obtained from database: https://bhichallenge.med.auth.gr/ICBHI_2017_Challenge. Bowel sounds audio files were obtained from Database: https://www.kaggle.com/datasets/robertnowak/bowel-sounds
